# Lipidomics Revealed Plasma Phospholipid Profile Differences between Deceased and Recovered COVID-19 Patients

**DOI:** 10.3390/biom12101488

**Published:** 2022-10-15

**Authors:** Neven Žarković, Biserka Orehovec, Bruno Baršić, Marko Tarle, Marta Kmet, Ivica Lukšić, Franz Tatzber, Willibald Wonisch, Elżbieta Skrzydlewska, Wojciech Łuczaj

**Affiliations:** 1 Ruđer Bošković Institute, Laboratory for Oxidative Stress, 10000 Zagreb, Croatia; 2 Clinical Hospital Dubrava, 10000 Zagreb, Croatia; 3 Department of Pathology, University of Zagreb School of Medicine, 10000 Zagreb, Croatia; 4 Omnignostica Ltd., 3421 Höflein an der Donau, Austria; 5 Department of Analytical Chemistry, Medical University of Bialystok, A. Mickiewicza 2D, 15-222 Bialystok, Poland

**Keywords:** COVID-19, SARS-CoV-2, lipidomics, phospholipids, plasma, low-density lipoprotein

## Abstract

Thorough understanding of metabolic changes, including lipidome alteration, associated with the development of COVID-19 appears to be crucial, as new types of coronaviruses are still reported. In this study, we analyzed the differences in the plasma phospholipid profiles of the deceased COVID-19 patients, those who recovered and healthy people. Due to identified abnormalities in plasma phospholipid profiles, deceased patients were further divided into two subgroups (D1 and D2). Increased levels of phosphatidylethanolamines (PE), phosphatidylcholines (PC) and phosphatidylserines (PS) were found in the plasma of recovered patients and the majority of deceased patients (first subgroup D1) compared to the control group. However, abundances of all relevant PE, PC and PS species decreased dramatically in the plasma of the second subgroup (D2) of five deceased patients. These patients also had significantly decreased plasma COX-2 activity when compared to the control, in contrast to unchanged and increased COX-2 activity in the plasma of the other deceased patients and recovered patients, respectively. Moreover, these five deceased patients were characterized by abnormally low CRP levels and tremendous increase in LDH levels, which may be the result of other pathophysiological disorders, including disorders of the immune system, liver damage and haemolytic anemia. In addition, an observed trend to decrease the autoantibodies against oxidative modifications of low-density lipoprotein (oLAb) titer in all, especially in deceased patients, indicate systemic oxidative stress and altered immune system that may have prognostic value in COVID-19.

## 1. Introduction

Coronaviruses are a large family of viruses known to cause colds and severe acute respiratory syndrome [1]. COVID-19 is caused by a new type of coronavirus-SARS-CoV-2, first reported in Wuhan in 2020 [2]. Over 547 million confirmed COVID-19 cases have been registered, and the disease claimed 6.3 million lives as reported on 3 July 2022 [3]. Although the early symptoms of COVID-19 infection are similar to influenza virus infection, most patients are prone to respiratory failure leading to interstitial lung lesions [4]. In addition, in severe cases, renal failure, coagulation disorders and sepsis may occur [4]. All the above-mentioned conditions accompanying COVID-19, especially in the elderly, contribute to the high COVID-19 mortality rate, which can be over 60% [5]. As the pathophysiology of the disease is still ambiguous, a detailed understanding of pathophysiological changes related to COVID-19 seems to be crucial for an effective diagnosis and pharmacotherapy.

One of the groups of biologically important compounds for the functioning of the organism is phospholipids, which constitute cell barriers and participate in their differentiation and apoptosis [6]. They also play an important role in signal transduction and energy storage. Changes in the composition and content of phospholipids may result from both changes in gene expression and the activity of enzymatic proteins involved in their biosynthesis and metabolism [7]. In contrast, abnormal phospholipid metabolism may contribute to the development of various metabolic diseases [8]. Therefore, the assessment of lipid metabolism disorders is very important in various pathological states, including metabolic disorders resulting from viral diseases. So far, significant changes in plasma lipid metabolism have been demonstrated in patients with various viral diseases, including Ebola virus and TBEV [9,10,11]. We have previously indicated that systemic oxidative stress manifested by pronounced lipoid peroxidation and vascular stress occurs in COVID-19 patients, especially in case of a lethal outcome, since the immune system of these patients is also malfunctioning [12]. Therefore, detailed lipidomic analyses were conducted to find if there are some specific changes in the lipid metabolism between the survivors and the deceased patients that could help in understanding the pathology of aggressive COVID-19.

## 2. Materials and Methods

The material for examinations was plasma collected from 20 (12 female and 8 male) COVID-19 recovered patients **[COVID-19 R]** with an average age of 65 years (54–85). Twenty COVID-19 deceased patients **[COVID-19 D]** have also participated in the study (12 female and 8 male), with an average age of 73 years (58–85). The **control group** consisted of 20 healthy subjects (12 female and 8 male) with an average age of 44 years (33–56) (Table 1).

It should be noted that, due to abnormalities in clinical parameters (CRP and LDH in particular), the group of deceased COVID-19 patients was divided into two subgroups (Table 2). The first subgroup **[COVID-19 D1]** included fifteen COVID-19 deceased patients (9 female and 6 male) with an average age of 72 years (58–85). Five COVID-19 deceased patients (3 female and 2 male) with an average age of 74 years (64–80) represented the second subgroup **[COVID-19 D2]**.

Blood samples were collected from patients treated at the Dubrava Clinical Hospital in Zagreb, which serves as the national COVID-19 center, thus providing medical care to patients suffering from COVID-19. This study was conducted after obtaining the approval of the ethics committee 2020-1012-13 of the Dubrava Clinical Hospital in Zagreb, while patients included in the study have signed informed consent. All patients who were able to sign informed consent upon arrival at the hospital were included in the study. Patients who were at terminal stage or suffering from any severe chronic disease such as cancer, diabetes or autoimmune disease that could affect the results were not enrolled in the studies. Before arriving at the hospital, none of the patients had been treated with drugs that could affect lipid metabolism or oxidative stress (like NSAIDs) and were not on any specific diet. However, due to the severity of the disease, they usually fasted.

All samples of blood were taken from the above-mentioned patients upon arrival to the hospital, i.e., before they received any therapy and days before the final outcome of their disease. Their blood, as well as the blood of healthy people, was taken into ethylenediaminetetraacetic acid (EDTA) tubes. The blood was centrifuged at 3000× *g* (4 °C) to obtain the plasma. Butylated hydroxytoluene (BHT) as an antioxidant was added to all samples to prevent oxidation. The samples were stored at −80 °C until analysis.

### 2.1. Extraction of Lipids and Quantification of Phospholipid Content

Lipid extracts from plasma samples were obtained using the modified Folch method [13]. The phospholipid content of each lipid extract was calculated by the colorimetric phosphorus assay [14]. All experimental procedures concerning lipid extraction and phospholipid quantification are described in detail in previously published studies [15,16].

### 2.2. Phospholipid Profiling by Hydrophilic Interaction Liquid Chromatography Coupled with High-Resolution Tandem Mass Spectrometry (Hilic-Ms/MS)

Phospholipids were separated by hydrophilic interaction liquid chromatography using a UPLC system (Agilent 1290; Agilent Technologies, Santa Clara, CA, USA) coupled with a QTOF mass spectrometer (Agilent 6540; Agilent Technologies, Santa Clara, CA, USA). Internal standards of PC(14:0/14:0), LPC(19:0), PE(14:0/14:0), PI(16:0/16:0) and PS(14:0/14:0) were used for the quantification and assessment of the ions variations. The mixture composed of solvent A [ACN/MeOH/water 50:25:25 (*v*/*v*/*v*) with 1 mM ammonium acetate] and solvent B [ACN/MeOH 60:40 (*v*/*v*) with 1 mM ammonium acetate] was used as mobile phase. The gradient elution was applied, starting with 0% of A, increased linearly to 100% of A within 20 min and held for 15 min, then returned to 0% of A in 10 min. Twenty-five μg of each phospholipid extract corresponding to a volume of 10 μL was mixed with 90 μL of mobile phase (60% of A and 40% of B). A volume of 10 μL of diluted sample was loaded into the Ascentis^®^ Si column (15 cm × 1 mm, 3 μm, Sigma-Aldrich, St. Louis, MO, USA) with the mobile phase flow rate of 40 μL per min. The QTOF mass spectrometer operated using a negative-ion mode (electrospray voltage, −3000 V) with capillary temperature of 250 °C and sheath gas flow of 13 L/min. Data-dependent acquisition mode (DDA) was used for data collecting in the range of *m*/*z* 100–1500 with a fixed collision energy of 35 eV. The LPE, PE, PI and PS species were analyzed as [M−H]^−^ ions, while LPC, PC and SM species were analyzed as [M+CH_3_COO]^−^ adducts. Data acquisition was carried out with the use of Mass Hunter data software (version B0.8.0, Agilent Technologies, Santa Clara, CA, USA). The relative content of each phospholipid ion species was achieved by normalization of the area of each peak to the peak area of the corresponding internal standard. The retention times and obtained MS/MS spectra were the basis for phospholipid identification.

### 2.3. Determination of COX-1 and COX-2 Activity

The activity of cyclooxygenase 1/2 and 2 (COX-1/2-EC.1.14.99.1) was examined spectrophotometrically using a commercial assay kit (Cayman Chemical Company, Ann Arbor, MI, USA) in accordance with the manufacturer’s instructions. COX 1 and 2 activity was measured spectrophotometrically (at 590 nm) by monitoring the appearance of oxidized N,N,N’,N’-tetramethyl-p-phenylenediamine (TMPD) [17]. One unit for COX-1 and COX-2 was defined as the amount of enzyme that will cause the oxidation of 1.0 nmol of TMPD per minute at 25 °C.

### 2.4. Determination of the Antibodies Directed against Oxidative Modifications of Low-Density Lipoprotein (Olab) Titer

The oLAb titer was determined by ELISAs using microtitration plates coated with oxidized LDL and blocked with bovine serum albumin. For the detailed experimental procedures of oLAb determination, the reader is referred to some previously published studies in which the same method was used [18,19].

### 2.5. Data Processing

The filtering, peak detection, alignment and integration as well as the assignment of each phospholipid species was carried out by the MZmine 2.30 software for the data obtained [20].

### 2.6. Statistical Analysis

Univariate and multivariate statistical analyses were performed using Metaboanalyst version 4.0 [21]. The data obtained by MS/MS analysis were autoscaled before Principal component analysis (PCA). Univariate statistical analysis was carried out using the ANOVA test with Tukey’s post hoc test with *p* < 0.05 considered statistically significant. The heatmaps were created using “Euclidean” as the clustering distance and “Ward” as the clustering algorithm.

## 3. Results

We analyzed 60 plasma samples, including three experimental groups: 20 healthy subjects **[Control],** 20 COVID-19 recovered patients **[COVID-19 R]** and 20 COVID-19 deceased patients **[COVID-19 D]** with the use of the HILIC-LC-MS/MS platform. We identified phospholipid species from PC, PE, phosphatidylinositols (PI), PS, lyso-phosphatidylethanolamines (LPE), lyso-phosphatidylcholines (LPC) and sphingomyelins (SM). The list of most abundant PL species which were identified and quantified are presented in Supplementary Table S2 (Supplementary Materials). Initially, we evaluated the obtained data from the whole set comprised of 60 plasma samples. PCA analysis was used to confirm the quality of the datasets and to visualize differences between groups of samples in the unsupervised analysis. Obtained PCA model (Supplementary Materials Figure S1) revealed high disperse of 20 plasma samples from deceased COVID-19 patients **[COVID-19 D]**.

**Figure 1:**
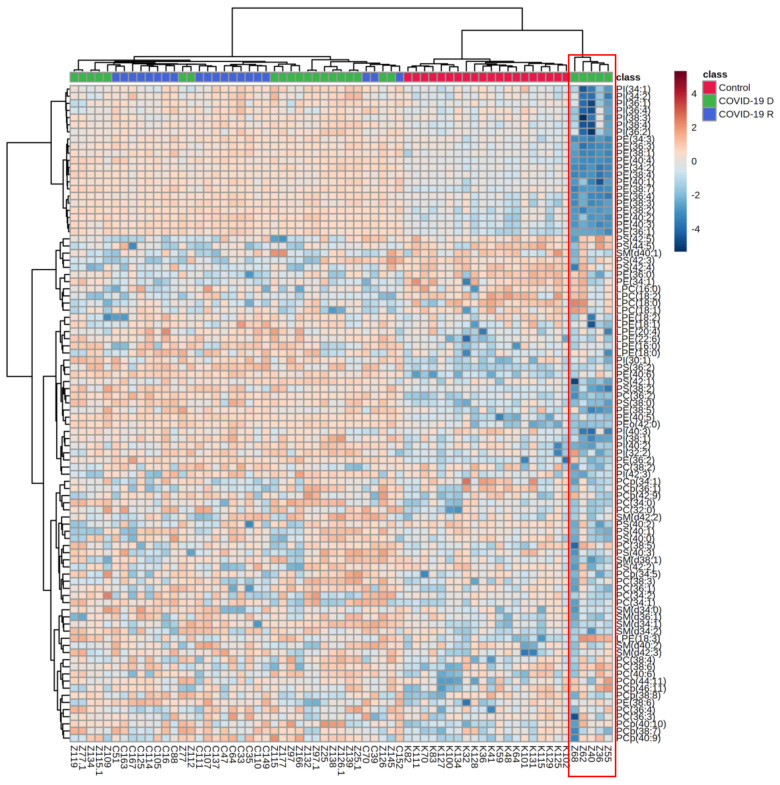
Two-dimensional hierarchical clustering heat map of the 50 most discriminating phospholipid species (according to One-way ANOVA) identified in the plasma of healthy subjects (*n* = 20) **[Control],** deceased COVID-19 patients (*n* = 20) **[COVID-19 D]** and COVID-19 recovered patients (*n* = 20) **[COVID-19 R]**.

Five plasma samples indicated by red ellipse on the Supplementary Figure S1 were responsible for observed dispersion within the **COVID-19 D group**. These samples did not cluster with the other fifteen plasma samples from the other deceased COVID patients. Moreover, we found an opposite direction of changes in the relative content of phospholipid species, particularly PI, PE and PS, in the plasma of these five deceased COVID-19 patients in comparison to the other 15 deceased COVID-19 patients (Figure 1).

**Figure 2:**
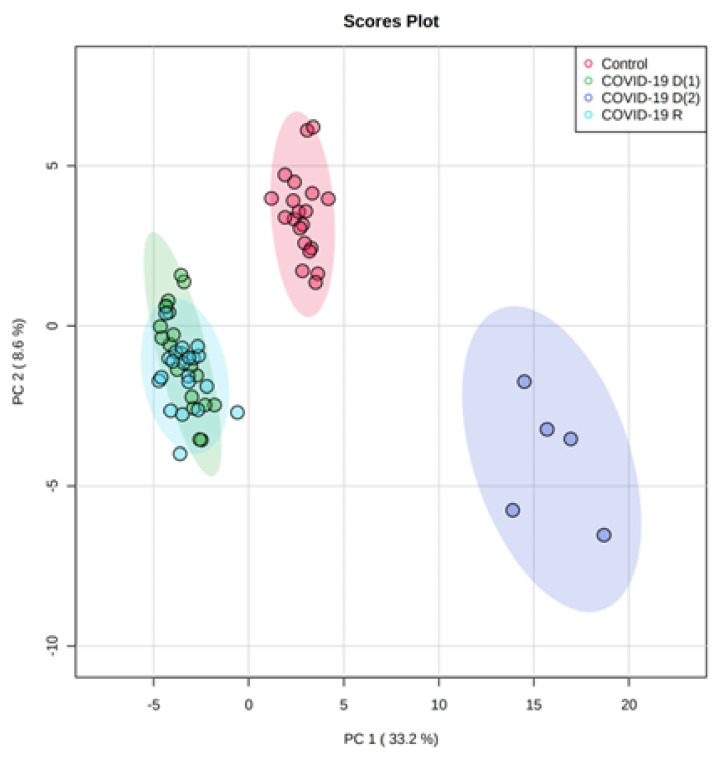
Two-dimensional principal component analysis (2D PCA) scores plot of the relative phospholipid content in the plasma of healthy subjects (*n* = 20) **[Control]**, first subgroup of deceased COVID-19 patients (*n* = 15) **[COVID-19 D(1)]**, second subgroup of deceased COVID patients (*n* = 5) **[COVID-19 D(2)]** and COVID-19 recovered patients (*n* = 20) **[COVID-19 R]**.

Therefore, considering the above-mentioned observations, we decided to split the group of deceased COVID-19 patients into two subgroups: the **COVID-19 D(1)** subgroup, including fifteen plasma samples, and the **COVID-19 D(2)** subgroup comprising five unusual plasma samples, giving finally four groups of samples for further analysis. The obtained PCA model for the final set of four groups (Figure 2) clearly shows that five plasma samples (indicated in purple) of deceased patients from the **COVID-19 D(2) subgroup** creates a cluster very well separated from the groups of healthy subjects and recovered COVID-19 patients **[COVID-19 R]**, but also from the plasma samples (indicated in green) from the other 15 deceased patients **[COVID-19 D(1) subgroup]**.

The model captured 41.8% of the total variance. The first principal component PC1, which accounted for almost 33.2%, describes the discrimination between samples from the **COVID-19 D(2)** subgroup scattered in the right region of the plot and the other analyzed three groups (**COVID-19 D(1)**, **COVID-19 R** and **Control**) scattered in the left region of the plot. The PC2 component accounted for 8.6% is responsible for the observed separation of control plasma samples from the COVID-19 recovered and both subgroups of deceased COVID-19 patients (**COVID-19 D(1)** and **COVID-19 D(2)**).

**Figure 3:**
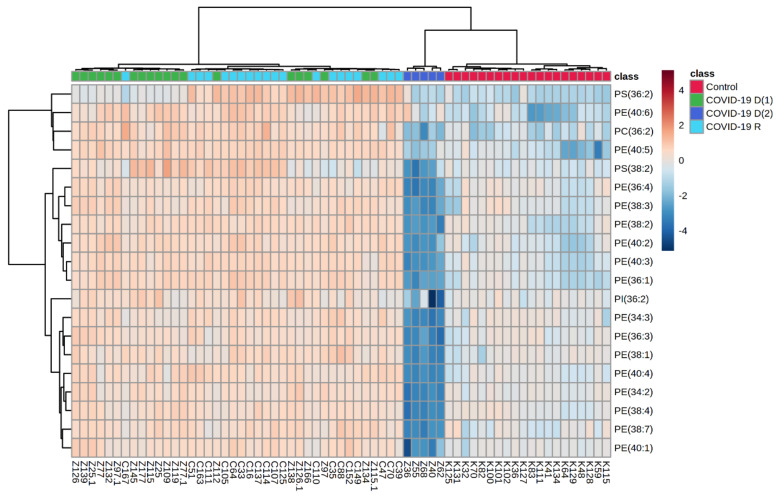
Two-dimensional hierarchical clustering heat map of the 20 most discriminating phospholipid species (according to One-way ANOVA) identified in the plasma of healthy subjects (*n* = 20) **[Control]**, first subgroup of deceased COVID-19 patients (*n* = 15) **[COVID-19 D(1)]**, second subgroup of deceased COVID patients (*n* = 5) **[COVID-19 D(2)]** and COVID-19 recovered patients (*n* = 20) **[COVID-19 R]**.

We also performed a univariate analysis in order to evaluate the variation in the relative abundances of phospholipid species belonging to each class. We sorted phospholipid molecular species using the p values from the One-way ANOVA test (Table 3, Figure 3).

The obtained results show that PE, PC and PS species were upregulated in the plasma of COVID-19 recovered patients compared with healthy subjects (Figure 3, Table 3). Upregulation of these three phospholipid classes and one PI specie was also observed in the plasma of the first subgroup of deceased COVID-19 patients (**COVID-19 D(1)**).

In contrast, a significant decrease in relative abundances of all mentioned phospholipid species was found in the plasma of deceased COVID patients belonging to the **COVID-19 D(2)** subgroup in comparison to healthy controls. Moreover, relative abundances of mentioned phospholipid species were also significantly downregulated in **COVID-19 D(2)** subgroup of deceased patients when compared to **COVID-19 D(1)** subgroup. While no significant changes were found between **COVID-19 D(1)** and COVID-19 recovered patients (**COVID-19 R**), a dramatic decrease of relative abundances of PE, PS, PI and PC was observed in the plasma of deceased COVID patients from **COVID-19 D(2)** group was responsible for observed their differentiation from COVID-19 recovered **COVID-19 R** (Figure 3, Table 2). In general, among all observed changes in relative abundances of phospholipid species, the most spectacular differences, concerning in particular downregulation of PE species, were found in the **COVID-19 D(2)** subgroup when compared to **COVID-19 D(1)** or **Control** groups. Therefore, in order to provide a better characterization of the **COVID-19 D(2)** group of samples, we compared its phospholipid profile with the **Control** group and **COVID-19 D(1)** group and performed multivariate and univariate analysis for both comparisons (Supplementary Materials Figures S2–S5).

We also examined the activities of COX-1 and COX-2 (Table 4), the enzymes involved in the oxidative metabolism of fatty acid.

The activities of COX-1 and COX-2 were significantly elevated in the plasma of recovered COVID-19 patients in comparison to healthy subjects. However, while the activity of COX-1 was elevated in the plasma of first subgroup of deceased COVID-19 patients (**COVID-19 D(1)**), no significant difference in the activity of this enzyme was found in the second subgroups of deceased patients (**COVID-19 D(2))**. In contrast, the activity of COX-2 was about three and two times lower in the plasma of deceased patients from the second subgroup as compared to the recovered patients and control, respectively.

**Figure 4:**
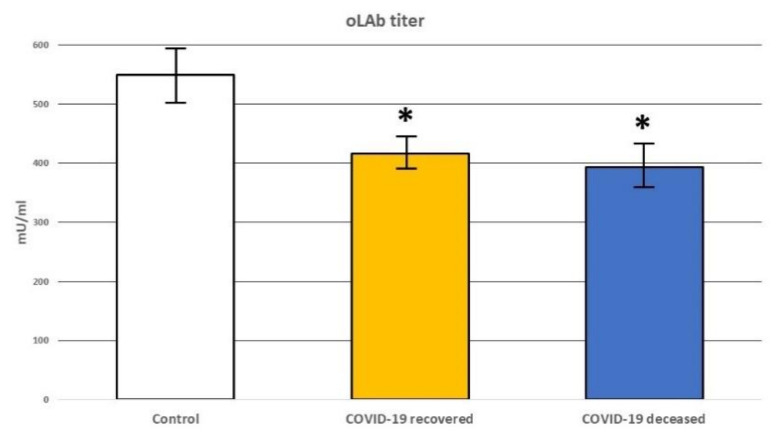
Changes in the level of antibodies directed against oxidative modifications of Low-Density Lipoprotein (oLAb) in the plasma of healthy subjects **[Control]** and recovered COVID-19 patients **[COVID-19 recovered]** and deceased COVID patients **[COVID-19 deceased]**. * means significant difference (*p* < 0.05) to the healthy control.

In order to evaluate the possible effects of lipid peroxidation on the immune reactivity of the patients, the titer of auto-antibodies (oLAb) directed against oxidative modifications of low-density lipoprotein (LDL) was also determined. Significantly decreased oLAb titer was observed in the plasma of both recovered COVID-19 patients and deceased COVID-19 with the trend to further reduction in the case of non-survivors when compared to healthy subjects, but this was not significant (Figure 4). Therefore, we can assume that the alterations of the lipid metabolism in COVID-19 patients resulted in peroxidation of the LDL that could result in the spread of systemic oxidative and vascular stress associated with the production of 4-hydroxynonenal (4-HNE), thus confirming our previous findings [12,22].

## 4. Discussion

Phospholipids are important structural components of cell membranes and energy storage molecules. Lipid mediators derived from phospholipids have been shown to play a crucial role in different metabolic pathways and cellular functions, particularly in inflammation and immunity [23,24]. Since alterations in plasma lipidome reflect systemic changes in the metabolism of different types of cells and organs, an assessment of the phospholipid composition and content in the plasma is very useful. Moreover, plasma phospholipid profiling allows the discrimination of bacteria from viral infection [25]. In recent years, a few studies focused on host lipid metabolism alteration in the response to COVID-19 have been published [26,27]. However, research data from studies on the phospholipidomic changes in COVID-19 recovered patients versus deceased COVID-19 patients is still limited. Therefore, we aimed to analyze differences and similarities in plasma phospholipid profiles of recovered COVID-19 patients, deceased COVID-19 patients and healthy volunteers. However, due to the opposite direction of changes in the relative content of phospholipid classes observed in this study and large differences in clinical parameters (especially CRP and LDH) between COVID-19 patients who have died, we decided to divide this group into two subgroups, including 15 and 5 patients in the first and second groups, respectively (Table 2). In this study, we found elevated levels of PE, PC and PS in the plasma of COVID-19 survivors compared to healthy controls. A similar direction of changes was observed in the vast majority of deceased COVID-19 patients (the first subgroup of 15 patients). However, the most interesting finding was that the relative abundances of all relevant PE, PC and PS species were dramatically decreased in the plasma of deceased COVID-19 patients from the second atypical subgroup (5 patients) when compared to the other 15 patients who died as well as patients who survived.

The majority of all relevant phospholipid species identified in this study belong to PE. It is well known that the accumulation of these phospholipids in the host cells causes significant inhibition of the inflammatory response induced by viruses [28]. Thus, observed in this study, the upregulation of PE in the plasma of COVID-19 survivors may confirm their involvement in the anti-inflammatory mechanisms. On the other hand, it can also be assumed that a significant decrease in the PE species found in the plasma of the above-mentioned five deceased COVID-19 patients led to a strong promotion of inflammatory processes, contributing to some extent to their poor response to therapy and, finally, death. Moreover, such a situation may also be exacerbated by age-related chronic comorbidities in these patients. This corresponds to the fact that PE-containing PUFAs were significantly and positively associated with clinical indicators of systemic inflammation [29]. It should be emphasized that the vast majority of the most relevant PE species contain linoleic acid (LA, 18: 2) or arachidonic acid (AA, 20: 4). Both polyunsaturated fatty acids, especially arachidonic acid and its metabolites, play a key role in the response to viral infection, including coronaviruses [30]. It is also important that LA and AA were recognized among other PUFAs, as the potential precursors for 4-HNE formation, which is a product of the oxidative fragmentation of the PUFAs hydrocarbon chain [31,32]. 4-HNE is a low-molecular α,β-unsaturated aldehyde that, as an electrophilic compound, can react with nucleophilic centers of other compounds, including proteins with adducts formation [33]. Recently formation of 4-HNE protein adducts was shown to be highly correlated with the severity of COVID-19. Moreover, it was also hypothesized that the formation of 4-HNE-proteins adducts contributed to the mechanisms that led to the death of COVID-19 patients [12,22]. 4-HNE has also been suggested to be involved in human pathologies since it is considered a key molecule in inflammation-related cell signaling [34]. It has been shown that under inflammatory conditions, the increased 4-HNE level leads to the upregulation of COX-2 and the initiation of the prostaglandin-dependent signaling pathways [35]. In this study, the activities of COX-1 and COX-2 were significantly elevated in the plasma of recovered COVID-19 patients in comparison to healthy subjects. However, while the activity of COX-1 was slightly elevated in the plasma of the first subgroup of deceased COVID-19 patients (COVID-19 D(1)), no significant difference in the activity of this enzyme was found in the second subgroups of deceased patients (COVID-19 D(2)). More interestingly, the activity of COX-2 was about three and two times lower in the plasma of deceased patients from the second subgroup as compared to the recovered patients and control, respectively. It is well known that both COX-1 and COX-2 are responsible for the production of lipid mediators involved in inflammation and immune responses [36]. These enzymes also mediate a variety of biological actions involved in vascular pathophysiology. Therefore, based on obtained results for COXs, we may speculate that the opposite direction of changes in phospholipids content observed in the plasma of these five unusual deceased patients is not only related to spreading the virus in their body but also associated with their response to inflammation that accompanied COVID-19 infection.

PS are the second group of relevant phospholipids responsible for observed in this study differences between deceased COVID-19 patients. As lipidomic alterations in patient plasma mainly reflect the systemic changes in the metabolism of diverse cell types and organs, we may assume that changes in PS relative content, observed in this study in the plasma of deceased and recovered COVID-19 patients, may be associated with inflammatory processes and coagulation. Several biochemical mechanisms that support the possible participation of PS in the inflammation and coagulation abnormalities in the pathophysiology of COVID-19 have been recently proposed [37]. Although it is well known that PS is involved in the entry of viruses, more recently, it has also been revealed that the presence of PE on the same surface of COVID-19 infected cells as PS dramatically enhances recognition by PS-binding proteins such as GAS6, PROS and TIM1, which are PS receptors involved in the cellular process of virus entry [38]. Moreover, it has been shown that in pathophysiological conditions, PS exposure may have a detrimental effect on coagulation activation and viral spread [39,40]. In this context, the PS exposure on the outer leaflet of the cell membrane, as a consequence of viral infection, has been suggested as another possible underlying mechanism of acute immune inflammatory response and activation of coagulation cascade [37]. It is well known that the damage to endothelial cells after viral infection and/or activation of endothelium by pro-inflammatory cytokines, such as IL-1β, IL-6, IFN-γ, IL-8 and TNF-α induces platelets and monocyte aggregation in the vascular wall. In addition, the intense inflammatory response could be involved in the formation of a thrombus. Thanks to research from the last decade, it has been revealed that there is a sophisticated connection between innate immunity, platelet activation and coagulation, termed immunothrombosis, which is an important host defense mechanism to limit the systemic spreading of pathogens through the bloodstream [41]. However, the aberrant activation of immunothrombosis may lead to myocardial infarction, stroke and pulmonary embolisms. Considering the above, it may be assumed that acute inflammation and coagulation abnormalities in COVID-19 patients may be one of the main causes of their death. Therefore, we may speculate that the other possible explanation of the opposite direction of changes in PL species observed in this study between both subgroups of deceased COVID patients may be associated with the interplay between inflammation and thrombosis.

The results obtained in this study suggest that infection with the SARS-CoV-2o virus may affect in a different way the systemic lipid metabolism of those unusually five deceased COVID-19 patients. It should also be noted that the duration of life in the Intensive Care Unit of these five patients was 6.2 days compared to the other patients, who died after 13.92 days (Table 1). The assessment of the basic diagnostic parameters of these five patients showed remarkably decreased values of C-reactive protein (CRP) and increased values of lactate dehydrogenase (LDH). On the other hand, it is believed that LDH and CRP may be related to respiratory function and serve as a predictor of respiratory failure in COVID-19 patients [42]. However, these five atypical patients did not show significant differences in oxygen saturation in relation to the other fifteen deceased patients enrolled in the study (Table 1). In contrast, lowered CRP levels may also suggest a malfunction of the immune system and possibly the liver, while elevated levels of LDH, a cellular enzyme released into the bloodstream, may be associated with a number of conditions, certainly including liver damage as well as hemolytic anemia [43,44,45]. There are a few case reports that implicate a potential relationship between COVID-19 and autoimmune hemolytic anemia (AIHA) [46,47] and the simultaneous onset of COVID-19 and autoimmune hemolytic anemia [48,49]. Although, it should be stressed that the association of increased LDH levels with AIHA is quite common [50]. In addition to these observations, considering the recently revealed close relationship of lipid peroxidation with the development of COVID-19 [12], we analyzed the level of autoantibodies against oxidized-LDL (oLAb), which play an important role in diseases associated with lipid peroxidation, e.g., liver disease, autoimmunological diseases, obesity, heart and circulatory failure. We found significantly reduced oLAb titer in COVID-19 patients, with a decisive trend to decrease in those patients who passed away. It has been previously suggested that increasing oLAb titers in patients with sepsis seemed to be wholesome while decreasing values observed in non-surviving patients indicated an overwhelming lipid peroxidation [19]. With respect to what is mentioned above, we may assume that oLAb titer may have prognostic value also in COVID-19 patients.

We believe that these results may be the basis for further studies on the pathogenesis of COVID-19. An opposite direction of changes in the relative content of phospholipid classes (PS and PE in particular) in the plasma of the subgroup of five deceased COVID-19 patients may be associated with other diseases and/or ineffective pharmacotherapy of these patients, consequently leading to their death. Nevertheless, further studies on a larger group of patients are undoubtedly necessary, in parallel with a careful analysis of the results of standard laboratory tests. This may contribute to the development of a panel of specific diagnostic tests and, consequently, to the development of targeted pharmacotherapy for COVID-19.

## 5. Conclusions

The results of our study indicate that systemic oxidative stress and an altered immune system might have prognostic value in COVID-19, especially for the prediction of possible lethal outcomes. That might also be relevant for a better understanding of the pathogenesis of altered inflammatory response to the SARS-CoV-2 infection, manifested by systemic lipid peroxidation causing reduction of the autoimmune antibodies against oxidized LDL. The observed relative abundances of all relevant PE, PC and PS species being decreased in the plasma of deceased COVID-19 patients from the atypical subgroup in comparison to the other deceased patients and survivors might be of particular interest for future research. However, more data from a higher number of patients are needed to confirm this, while we believe that our results may be the basis for further studies on the pathogenesis of COVID-19.

## Figures and Tables

**Table 1:** Characteristics of COVID-19 deceased **[COVID-19 D]** and COVID-19 recovered patients **[COVID-19 R]** compared to healthy subjects **[Control]**.

Participants	Number of Participants	Sex (Female/Male)	Age Range (Average)	CRP [mg/L]	LDH [U/L]	Oxygen Saturation [%]
**Control** [healthy subjects]	20	12/8	33−56 (44)	0.00−5.00	140−280	> 95%
**COVID-19 D** [COVID-19 deceased patients]	20	12/8	58−85 (73)	132.3 ± 54.8	488.4−156.5	82.35 ± 14.05
**COVID-19 R** [COVID-19 recovered patients]	20	12/8	54−85 (65)	114.5 ± 61.8	355.0 ± 147.5	90.21 ± 6.05

**Table 2:** Characteristics of two subgroups of deceased COVID-19 patients **[COVID-19 D1]** and **[COVID-19 D2]**.

Participants	Number of Participants	Sex (Female/Male)	Age Range (Average)	CRP [mg/L]	LDH [U/L]	Oxygen Saturation [%]
**COVID-19 D1** [COVID-19 deceased patients subgroup 1]	15	9/6	58–85 (72)	168.2 ± 73.1	300.3 ± 145.9	81.4 ± 15.5
**COVID-19 D2** [COVID-19 deceased patients subgroup 2]	5	3/2	64–80 (74)	96.4 ± 36.6	676.5 ± 167.0	83.3 ± 12.6

**Table 3:** The alteration observed in the molecular species of 20 most discriminating (according to One-way ANOVA) phospholipid species from PE, PS and PI class in the plasma of healthy subjects (*n* = 20) **[Control]**, first subgroup of deceased COVID-19 patients (*n* = 15) **[COVID-19 D(1)]**, second subgroup of deceased COVID patients (*n* = 5) **[COVID-19 D(2)]** and recovered COVID-19 patients (*n* = 20) **[COVID-19 R]**.

PL Class	PL Specie	Phospholipid Molecular Species	Log_2_ (Fold-Change)					
			COVID-19 R vs. Control	COVID-19 D(1) vs. Control	COVID-19 D(2) vs. Control	COVID-19 D(2) vs. COVID-19 D(1)	COVID-19 D(1) vs. COVID-19 R	COVID-19 D(2) vs. COVID-19 R
PS	PS(36:2)	PS(18:0/18:2)	5.18 ↑	4.27 ↑	-	3.52 ↓	-	4.43 ↓
PE	PE(40:6)	PE(18:0/22:6)	3.64 ↑	3.29 ↑	2.46 ↑	-	-	1.19 ↓
PC	PC(36:2)	PC(18:0/18:2)	3.06 ↑	2.67 ↑	2.70 ↓	5.37 ↓	-	5.75 ↓
PE	PE(40:5)	PE(18:0/22:5)	3.15 ↑	3.22 ↑	-	-	-	4.02 ↓
PS	PS(38:2)	PS(20:0/18:2)	1.28 ↑	1.31 ↑	2.14 ↓	3.45 ↓	-	3.42 ↓
PE	PE(36:4)	PE(16:0/20:4)	1.71 ↑	1.71 ↑	8.16 ↓	9.86 ↓	-	9.87 ↓
PE	PE(38:3)	PE(20:0/18:3)	1.79 ↑	1.63 ↑	6.65 ↓	8.28 ↓	-	8.44 ↓
PE	PE(38:2)	PE(20:0/18:2)	1.83 ↑	1.74 ↑	5.67 ↓	7.41 ↓	-	7.49 ↓
PE	PE(40:2)	PE(18:0/22:2)	2.42 ↑	2.38 ↑	5.11 ↓	7.49 ↓	-	7.53 ↓
PE	PE(40:3)	PE(20:1/18:2)	1.54 ↑	2.92 ↑	7.96 ↓	10.88 ↓	-	11.09 ↓
PE	PE(36:1)	PE(18:0/18:1)	3.57 ↑	3.24 ↑	7.61 ↓	10.85 ↓	-	11.18 ↓
PI	PI(36:2)	PI(18:0/18:2)	-	1.29 ↑	2.45 ↓	3.74 ↓	-	3.42 ↓
PE	PE(34:3)	PE(16:1/18:2)	1.45 ↑	1.02 ↑	7.48 ↓	8.50 ↓	-	8.93 ↓
PE	PE(36:3)	PE(18:1/18:2)	1.32 ↑	1.27 ↑	6.66 ↓	7.93 ↓	-	7.98 ↓
PE	PE(38:1)	PE(20:0/18:1)	1.34 ↑	1.11 ↑	5.51 ↓	6.62 ↓	-	6.85 ↓
PE	PE(40:4)	PE(20:0/20:4)	2.32 ↑	2.30 ↑	8.39 ↓	10.69 ↓	-	10.71 ↓
PE	PE(34:2)	PE(16:0/18:2)	1.44 ↑	1.86 ↑	8.86 ↓	10.72 ↓	-	10.30 ↓
PE	PE(38:4)	PE(18:0/20:4)	1.43 ↑	1.77 ↑	9.39 ↓	11.17 ↓	-	10.82 ↓
PE	PE(38:7)	PE(16:1/22:6)	1.27 ↑	1.31 ↑	5.67 ↓	6.98 ↓	-	6.95 ↓
PE	PE(40:1)	PE(18:0/22:1)	1.71 ↑	1.55 ↑	5.46 ↓	7.04 ↓	-	7.16 ↓

↑: increase, ↓: decrease.

**Table 4:** Changes in the activity of COX-1 and COX-2 in the plasma of healthy subjects (*n* = 20) **[Control]**, first subgroup of deceased COVID-19 patients (*n* = 15) **[COVID-19 D(1)]**, second subgroup of deceased COVID patients (*n* = 5) **[COVID-19 D(2)]** and recovered COVID-19 patients (*n* = 20) **[COVID-19 R]**.

Groups	COX 1 [nmol/min/mL]	COX 2 [nmol/min/mL]
**Control**	14.59 ± 4.99	9.90 ± 1.90
**COVID-19 D1**	18.42 ± 3.61 ^ab^	14.23 ± 1.12 ^b^
**COVID-19 D2**	14.74 ± 2.74	5.71 ± 0.46 ^abc^
**COVID-19 R**	24.52 ± 3.22 ^a^	19.53 ± 4.14 ^a^

Mean values ± SD are presented with statistically significant differences: ^a^ means vs. control group; ^b^ means vs. COVID-19 R group; ^c^ means COVID-19 D1 subgroup, *p* < 0.05.

## Data Availability

The data presented in this study are contained within the article.

## Supplementary Materials

The following supporting information can be downloaded at: https://www.mdpi.com/article/10.3390/biom12101488/s1, Table S1: Relative content (related to internal standard) of each phospholipid species identified in the plasma of healthy subjects (*n* = 20) [Control], COVID-19 recovered patients (*n* = 20) [COVID-19 R], first subgroup of deceased COVID-19 patients (*n* = 15) [COVID-19 D(1)] and second subgroup of deceased COVID patients (*n* = 5) [COVID-19 D(2)]. Data obtained using MZmine software (XLSX); Table S2: LC-MS parameters of most abundant phospholipid species identified in the plasma of healthy subjects (*n* = 20) [Control], COVID-19 recovered patients (*n* = 20) [COVID-19 R], first subgroup of deceased COVID-19 patients (*n* = 15) [COVID-19 D(1)] and second subgroup of deceased COVID patients (*n* = 5) [COVID-19 D(2)]. Phosphatidylcholine (PC), lyso-phosphatidylcholine (LPC), phosphatidylethanolamine (PE), lyso-phosphatidylethanolamine (LPE), phosphatidylinositols (PI), phosphatidylserine (PS) and sphingomyelin (SM); Figure S1: Two-dimensional principal component analysis (2D PCA) scores plot of the relative phospholipid content in the plasma of healthy subjects (*n* = 20) [Control], deceased COVID-19 patients (*n* = 20) [COVID-19 D] and recovered COVID-19 patients (*n* = 20) [COVID-19 R]; Figure S2: Two-dimensional principal component analysis (2D PCA) scores plot of the relative phospholipid content in the plasma of 15 deceased COVID-19 patients [COVID-19 (D1)] and another group of 5 deceased COVID patients [COVID-19 (D2)]; Figure S3: Two-dimensional hierarchical clustering heat map of the 20 most discriminating phospholipid species (according to One-way ANOVA) identified in the plasma of 15 deceased COVID-19 patients [COV1 mort] and another group of 5 deceased COVID patients [COV2 mort]; Figure S4: Two-dimensional principal component analysis (2D PCA) scores plot of the relative phospholipid content in the plasma of 20 healthy subjects [Control] and 5 deceased COVID patients [COVID-19 (D2)]; Figure S5: Two-dimensional hierarchical clustering heat map of the 20 most discriminating phospholipid species (according to One-way ANOVA) identified in the plasma of 20 healthy subjects [Control] and 5 deceased COVID patients [COVID-19 (D2)].
