# Supplementary material for: Lipidomics Revealed Plasma Phospholipid Profile Differences between Deceased and Recovered COVID-19 Patients
Source: Biomolecules. 2022 Oct 15;12(10):1488. doi: 10.3390/biom12101488 (PMC9599609; doi:10.3390/biom12101488)
Supplement: Supplementary file 1 [file biomolecules-12-01488-s001.zip › biomolecules-1902778-supplementary.pdf]

Table S1. Relative content (related to internal standard) of each phospholipid species identified in the plasma of healthy subjects (n=20) [Control], COVID-19 recovered patients (n=20) [COVID-19 R], first subgroup of deceased COVID-19 patients (n=15) [COVID-19 D(1)] and second subgroup of deceased COVID patients (n=5) [COVID-19 D(2)].. Data obtained using MZmine software (XLSX).

	A	B	C	D	E	F	G	H	I	J	K	L	M
1	Name	K32	K36	K41	K48	K59	K64	K70	K82	K83	K100	K101	K115
2	Label	Control	Control	Control	Control	Control	Control	Control	Control	Control	Control	Control	Control
3	PC(34:2)	234154.6	370894	237372	220788.5	283520	249435.5	273299	263737.7	268406.7	259537	153638.2	312348.3
4	PC(34:1)	50737.04	148650.2	103473.9	175947.2	166556.2	74578.72	58801.92	79759.93	58420.97	156970.5	200042.8	243093.4
5	PC(36:4)	212762.2	59783.96	7707.011	101983.5	176381.2	59658.49	47881.76	94803.72	124954.8	74061.42	124331.3	182074.1
6	PCp(38:8)	27155.75	20554.75	63340.58	34417.82	21980.2	17199.33	3778.235	2739.278	2225.3	21033.42	12816.6	40109.25
7	PCp(34:5)	232802.6	131398.1	182960.3	212804.6	226196.6	120261.2	17267.86	100460.3	155251	103328.9	94849.1	166390.5
8	PC(36:3)	37568.82	56360.75	58708.81	75570.37	61908.42	73690.19	126101.8	61559.79	16155.62	68778.24	54965.63	93800.03
9	PC(38:4)	144034.8	32782.85	5691.731	22944.23	44057.53	45737.83	33368.41	37620.46	78910.97	55667.96	49366.03	105404.8
10	PC(36:2)	36064.73	22265.59	19866.29	45644.07	21976.1	14119.83	2291.902	4079.95	5148.302	3707.043	14083.13	5318.509
11	PCp(40:10	549.3145	1643.846	1521.535	3017.305	8942.307	3769.579	1974.124	860.2846	2882.558	1904.665	1865.871	6201.962
12	PC(38:3)	47505.23	25311.45	5180.241	6672.004	51923.28	21976.72	15164.81	6658.69	13160.61	37616.14	10692.99	44007.9
13	PC(36:1)	19444.46	72694.94	86383.26	35531.41	59844.38	22014.38	17267.89	35672.62	17649.53	60064.29	69563.35	80594.42
14	PC(38:6)	64211.54	41270.34	4904.579	10386.65	19095.66	29423.45	3314.064	9863.203	9878.442	2594.25	35163.53	58776.9
15	PCp(38:7)	26229.53	2018.489	5805.965	5353.53	14821.21	8590.286	1650.401	4515.497	2235.408	5262.661	7847.914	15701.97
16	PC(34:0)	44435.88	12468.88	10785.21	19143.83	20451.19	17080.99	7190.729	3658.983	28682.15	3110.879	5321.561	17288.14
17	PCp(34:1)	125331.1	53424.44	43086.61	22842.32	19485.07	4203.929	10205.45	4243.54	23457.3	6994.356	4103.596	2493.988
18	PCp(36:1)	46898.77	24725.43	23150.58	24683.3	9970.051	10744.87	12416.8	1579.659	6720.557	645.203	14260.04	1627.574
19	PC(38:5)	20111.37	10366.14	4966.059	9396.373	34875.3	6752.502	12220.92	8319.662	21073.86	12710.11	3805.413	38497.48
20	PCp(40:9)	8708.471	2936.749	1144.964	1699.987	8224.189	4152.603	3839.619	3721.747	4584.662	2037.231	12673.16	16652.65
21	PC(40:6)	10423.85	17722.72	6707.072	6618.036	13246.56	1238.706	13823.89	1262.107	3672.33	606.8678	641.0033	30295.16
22	PC(38:2)	7689.371	12020.72	10503.1	19520.88	2828.263	16585.78	22791.7	9588.926	12143.12	11201.96	1643.453	3706.661
23	PCp(44:11	5861.3	7104.222	927.7867	2320.17	16112.38	7062.567	6674.096	8380.26	4386.251	456.8641	1256.171	6945.923
24	PCp(46:11	1798.662	7662.382	620.689	3107.517	14362.43	1157.514	1907.727	274.7455	3618.661	290.2235	4024.983	9046.153
25	PC(32:0)	30477.23	10785.46	18368.98	19648.06	9226.216	11380.82	8735.598	4128.422	10211.89	802.2595	14369.07	4938.378
26	PCp(42:9)	8848.973	13334.88	27629.96	14096.61	29932.35	22657.6	3226.103	6954.919	12414.31	4242.667	9107.172	2718.069
27	LPC(16:0)	30176.83	67600.01	96787.5	172312.4	119962.9	84987.41	228190.2	95199.92	79164.41	99205.57	93510.1	62204.9
28	LPC(18:0)	25558.35	42365.05	53782.08	21320.21	40517.14	24262.99	6808.33	14823.05	4533.438	40789.29	32198.08	36166.74
29	LPC(18:1)	557.8825	14178.29	28781.43	13486.03	5532.576	16189.08	18827.39	10196.34	10364.91	9307.785	10792.1	10041.38
30	LPC(18:2)	25982.58	12603.87	40898.5	45132.28	25855.29	27127.05	16634.72	13630.33	20127.19	8630.318	30220.27	3714.928
31	PI(38:4)	84493.51	122922.9	69497.36	96191.08	33513.43	98844.35	91244.95	88982.49	35549.97	47177.44	177744.7	66486.32
32	PI(36:2)	30052.3	30759.41	16453.44	29715.69	27466.5	25353.56	23812.23	32884.49	18969.65	21196.95	48893.33	15862.36
33	PI(30:1)	1344.474	691.6931	2015.093	446.5514	361.8489	2195.633	1706.851	1685.864	691.8699	1849.831	7910.707	13521.07
34	PI(38:3)	22386.44	34421.9	15596.2	38123.25	22821.24	22870.94	26577.13	15919.99	25208.27	17998.3	37307.85	11119.36
35	PI(36:1)	8226.61	5763.976	10855.47	6007.077	4255.79	6496.682	5190.014	14369.22	8120.434	8452.989	9073.274	8823.396
36	PI(34:1)	7293.697	15988.13	8913.182	14515.34	6539.315	11036.83	9170.536	8040.707	7363.132	7023.061	31152.21	8270.89
37	PI(42:3)	1651.006	2318.802	3879.216	6008.05	3772.465	3522.001	14032.42	7796.232	8815.423	8247.45	4790.487	9110.662
38	PI(34:2)	7078.876	13835.41	13387.1	11779.92	4965.09	12111.78	6449.167	13836.25	5699.844	2687.73	9130.544	8110.257
39	PI(36:4)	6343.664	12552.47	11253.41	11253.71	10476.92	5293.938	7339.938	5182.206	9369.436	4201.561	19241.28	5719.641

	A	N	O	P	Q	R	S	T	U	V	W	X	Y	Z
1	Name	K125	K127	K128	K131	K102	K111	K129	K134	Z97	Z126	Z115	Z25	Z77
2	Label	Control	Control	Control	Control	Control	Control	Control	Control	COVID-19	COVID-19	COVID-19	COVID-19	COVID
3	PC(34:2)	549888.8	117460.9	451124.8	437654.9	154801.9	321760	377941.4	241369.4	172230.9	950220.1	123140.2	135853.5	2757
4	PC(34:1)	275650.8	62914.77	297508.9	166488.3	95603.28	96509.52	264971.8	196213.1	59218.05	609447.2	49761.6	51457.01	3183
5	PC(36:4)	120004	15805.42	65017.4	74132.12	106181.5	112816.4	207564.5	79986.34	102532.1	48938.15	318797.4	96187.11	6159
6	PCp(38:8)	33326.93	1798.771	24294.34	20143.66	13385.94	3232.348	47328.91	24819.43	6076.753	6293.603	18828.7	8410.237	6498
7	PCp(34:5)	156713.9	65272.93	181351.4	120886.2	145670.6	93428.04	186357.4	115728.3	251159.4	170163.2	66225.22	270583.5	2676
8	PC(36:3)	73733.76	18673.59	37704.91	63687.65	39869.02	56635.01	116312	85285.02	68830.54	20659.2	62997.38	56366.31	5790
9	PC(38:4)	89405.23	22046.17	9081.544	39667.25	28681.45	34610.82	98026.44	68471.59	238858.3	77772.29	17669.9	122390.2	1531
10	PC(36:2)	25732.68	26750.58	7812.56	24269.49	15075.6	3549.557	6648.136	4522.592	77767.53	86088.45	47211.88	91003.01	1147
11	PCp(40:10	7673.985	2920.088	1806.084	1873.985	6760.384	1075.356	6698.119	2514.158	32666.21	6742.943	3352.713	35571.19	1872
12	PC(38:3)	31552	11932.49	1939.18	23286.54	39617.47	8256.776	43127.74	34606.85	69356.22	107535.5	18292.03	70361.38	4107
13	PC(36:1)	36792.27	16273.82	38320.8	102499.9	46917.99	43877.32	74146.87	84690.65	169905.4	145835.1	109121.2	91620.04	152
14	PC(38:6)	25928.76	8769.294	30322.57	47873.59	15104.67	10750.89	66417.9	3009.33	63076.38	21323.45	3106.413	56371.64	1118
15	PCp(38:7)	11731.33	941.5392	26585.31	2482.741	11827.32	4876.736	18685.34	6262.566	15753.82	2772.63	22423.74	15552.55	4489
16	PC(34:0)	19118.74	11268.68	42163.83	15212.03	16606.37	3585.803	14867.8	2675.356	105227.9	80949.93	71242.63	60800.87	197
17	PCp(34:1)	11875.87	9789.66	4311.104	70520.26	16094.67	3649.444	2169.77	6085.09	10942	17836.94	1492.212	9745.635	1336
18	PCp(36:1)	4370.108	671.5421	4582.489	29423.26	8305.052	1295.32	2050.743	632.2989	16482.92	37347.09	850.1514	19609.56	512
19	PC(38:5)	29682.51	8254.946	7620.401	8914.882	29539.38	6489.336	31567.94	11693.3	23144.87	35350.98	3817.272	19881.41	9792.
20	PCp(40:9)	5873.328	1730.91	5421.334	2554.971	7023.458	5247.663	12989.06	2302.071	19064.81	4498.387	69753.3	15974.38	2087
21	PC(40:6)	1662.919	400.2582	10855.9	22330.63	11405.29	1665.981	27871.54	734.31	31556.33	7368.959	40862.3	18732.63	1195
22	PC(38:2)	23319.48	3466.877	24443.78	9856.992	2454.932	12082.05	5226.392	12210.14	4805.431	9540.169	12247.86	5819.012	1860
23	PCp(44:11	2972.786	437.4963	497.9919	5541.293	14098.34	9721.102	8057.271	520.8251	5938.915	5095.262	6293.533	10036.63	2070
24	PCp(46:11	3028.388	122.5515	1281.702	7126.015	12667.67	313.2098	11126.77	365.6816	1455.487	2857.76	5338.124	2499.998	428.4
25	PC(32:0)	26402.78	4203.159	19797.31	13481.83	8525.024	4665.116	6024.821	657.8528	9834.714	16978.58	54132.99	13911.13	9055.
26	PCp(42:9)	16205.33	2680.962	26698.33	14401.67	29543.23	7789.51	3587.85	3309.28	11585.03	53642.31	14587.57	12312.59	2142
27	LPC(16:0)	118810.4	28460.24	5156.585	93571.04	81300.73	120038.7	57850.56	79768.01	58852.55	50687.47	76977.21	19271.13	525
28	LPC(18:0)	33581.59	10046.95	21652.7	33224.06	12555.13	44460.33	45208.42	47448.86	8250.099	11451.41	21331.48	9484.929	1536
29	LPC(18:1)	17926.36	11735.57	3101.973	4758.015	8493.551	10610.87	10844.69	17581.08	2856.179	8329.298	7932.082	1969.145	9309.
30	LPC(18:2)	46467.69	19072.77	2794.855	22494.11	11258.65	10183.77	4383.615	12351.79	5267.49	1015.816	4876.055	5530.973	1350.
31	PI(38:4)	35632.81	52752.76	53830.44	30832.36	57927.6	52838.73	74464.67	113089	173941.5	129341.5	286702	233674	154
32	PI(36:2)	14320.13	10533.6	20496.84	25269.18	21177.61	26284.22	19669.33	34758.13	27444.53	35109.34	64559.27	115137.6	4968
33	PI(30:1)	10479.13	7233.677	5858.383	336.5195	1085.696	1720.342	16630.92	836.9487	32269.12	18963.44	3030.815	9615.46	2683
34	PI(38:3)	22542.67	12502.33	9149.826	22364.81	9695.273	22497.88	13565.61	37519.87	43092.56	19136.4	30095.71	43896.35	2596
35	PI(36:1)	9159.012	4318.777	17032.53	4596.254	12573.07	9129.228	11646.88	6570.932	2849.513	11879.51	25310.61	11158.26	1611
36	PI(34:1)	4390.634	3961.669	12151.87	7127.854	6979.334	6882.6	7609.219	14709.08	10352.92	8612.08	31152.99	19738.65	8141.
37	PI(42:3)	1056.049	1910.74	12489.87	4225.161	6712.556	7587.654	12846.03	3269.511	4751.579	1203.9	12634.36	9958.492	1712.
38	PI(34:2)	2675.095	5805.483	6583.368	5610.551	10557.06	3037.135	9407.899	16049.08	11922.56	5374.376	45912.42	13218.5	6344.
39	PI(36:4)	7138.859	3071.676	3979.009	11943.69	3917.748	4999.858	6806.373	15439.54	5239.585	7694.761	11942.83	10700.49	9719.

NEW (2) All PL COV D1(15) and D

Table S2. LC-MS parameters of most abundant phospholipid species identified in the plasma of healthy subjects (n=20) [Control], COVID-19 recovered patients (n=20) [COVID-19 R], first subgroup of deceased COVID-19 patients (n=15) [COVID-19 D(1)] and second subgroup of deceased COVID patients (n=5) [COVID-19 D(2)]. Phosphatidylcholine (PC), lyso-phosphatidylcholine (LPC), phosphatidylethanolamine (PE), lyso-phosphatidylethanolamine (LPE), phosphatidylinositols (PI), phosphatidylserine (PS), and sphingomyelin (SM).

Phospholipid class	m/z	Retention time	Phospholipid specie
**PC**	816.5771	15.94	PC(34:2)
	818.5877	16.00	PC(34:1)
	840.5747	15.78	PC(36:4)
	844.5501	16.11	PCp(38:8)
	794.5308	15.84	PCp(34:5)
	842.5914	15.92994	PC(36:3)
	868.6059	15.9614	PC(38:4)
	844.6037	18.33565	PC(36:2)
	868.5497	15.99892	PCp(40:10)
	870.6181	16.08245	PC(38:3)
	846.6186	16.19802	PC(36:1)

	864.5705	15.8714	PC(38:6)
	846.5652	16.3295	PCp(38:7)
	820.6088	16.46466	PC(34:0)
	802.5923	17.15636	PCp(34:1)
	830.6293	16.87348	PCp(36:1)
	866.5874	15.90546	PC(38:5)
	870.5732	16.2772	PCp(40:9)
	892.608	16.67807	PC(40:6)
	872.638	16.71384	PC(38:2)
	922.5956	15.87104	PCp(44:11)
	950.6242	16.10888	PCp(46:11)
	792.5763	16.88813	PC(32:0)
	898.5951	16.14108	PCp(42:9)
**LPC**	554.3458	21.5818	LPC(16:0)
	582.3725	21.05616	LPC(18:0)
	580.3587	21.1634	LPC(18:1)
	578.3486	21.30834	LPC(18:2)
**PI**	885.5456	4.821439	PI(38:4)
	861.5454	4.910499	PI(36:2)
	779.4705	4.561882	PI(30:1)
	887.5668	4.886841	PI(38:3)
	863.5657	4.897894	PI(36:1)
	835.5302	4.963673	PI(34:1)
	943.6283	4.541324	PI(42:3)
	833.5207	4.935864	PI(34:2)
	857.5172	4.909505	PI(36:4)
	915.5954	5.08065	PI(40:3)
	891.5935	5.564356	PI(38:1)
	917.6096	5.140741	PI(40:2)
	805.4846	4.654245	PI(32:2)
**SM**	761.58	18.07767	SM(d34:1)
	871.6844	17.89147	SM(d42:2)
	789.6111	18.18377	SM(d36:1)
	843.6527	17.93301	SM(d40:2)
	845.6746	18.04331	SM(d40:1)
	817.6417	17.92183	SM(d38:1)
	869.6728	18.03865	SM(d42:3)
	763.5977	18.44771	SM(d34:0)
	759.565	18.45793	SM(d34:2)
**PS**	814.5771	14.70518	PS(38:2)
	818.5878	14.65117	PS(38:0)

	840.5745	14.20162	PS(40:3)
	844.6037	14.43607	PS(40:1)
	868.6058	14.14999	PS(42:3)
	842.5914	14.40098	PS(40:2)
	786.4718	16.74137	PS(36:2)
	872.638	15.74979	PS(42:1)
	870.6178	14.3517	PS(42:2)
	846.6183	14.55719	PS(40:0)
	864.57	14.09764	PS(42:5)
	866.587	14.17848	PS(42:4)
	892.608	14.0946	PS(44:5)
**PE**	796.5541	13.74943	PE(40:3)
	744.572	13.73649	PE(36:1)
	794.5582	13.22917	PE(40:4)
	738.506	13.52855	PE(36:4)
	714.5886	13.01041	PE(34:2)
	766.5712	13.54073	PE(38:4)
	742.5878	14.35425	PE(36:2)
	746.5048	12.31981	PE(36:0)
	800.554	12.96447	PE(40:1)
	770.5139	13.76467	PE(38:2)
	712.5991	13.02382	PE(34:3)
	768.6005	13.41937	PE(38:3)
	740.5702	13.07924	PE(36:3)
	798.5826	13.61915	PE(40:2)
	760.5872	12.836	PE(38:7)
	762.5054	13.07883	PE(38:6)
	792.5567	13.61865	PE(40:5)
	716.5242	12.13795	PE(34:1)
	764.5421	13.45612	PE(38:5)
	772.5862	12.92291	PE(38:1)
	790.5402	13.14652	PE(40:6)
	816.6865	13.61444	PEo(42:0)
**LPE**	452.2765	11.30476	LPE(16:0)
	500.2737	9.306383	LPE(20:4)
	480.3056	9.637998	LPE(18:0)
	524.2747	9.409675	LPE(22:6)
	476.2799	9.913107	LPE(18:2)
	478.2943	9.549429	LPE(18:1)
	474.2623	7.576531	LPE(18:3)

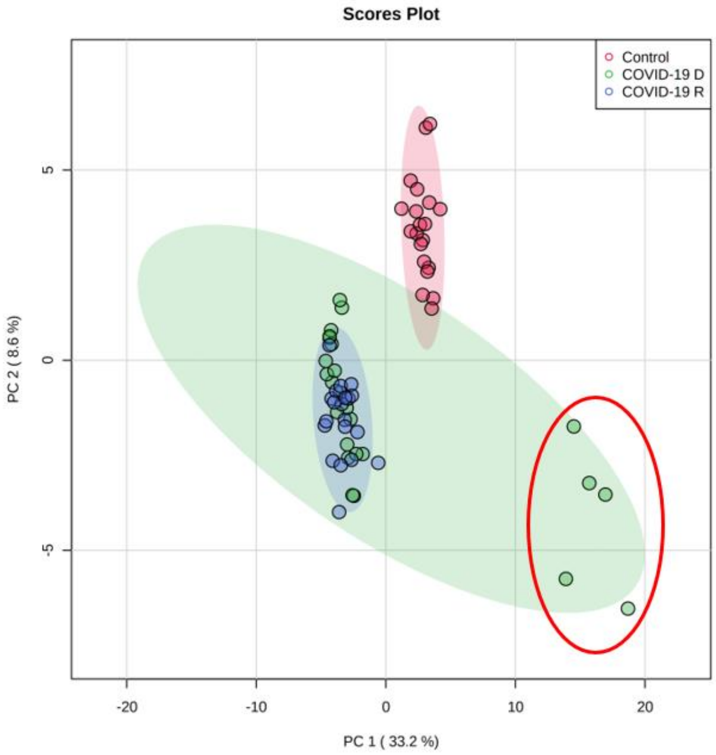

Figure S1. Two-dimensional principal component analysis (2D PCA) scores plot of the relative phospholipid content in the plasma of healthy subjects (n=20) **[Control]**, deceased COVID-19 patients (n=20) **[COVID-19 D]** and recovered COVID-19 patients (n=20) **[COVID-19 R]**.

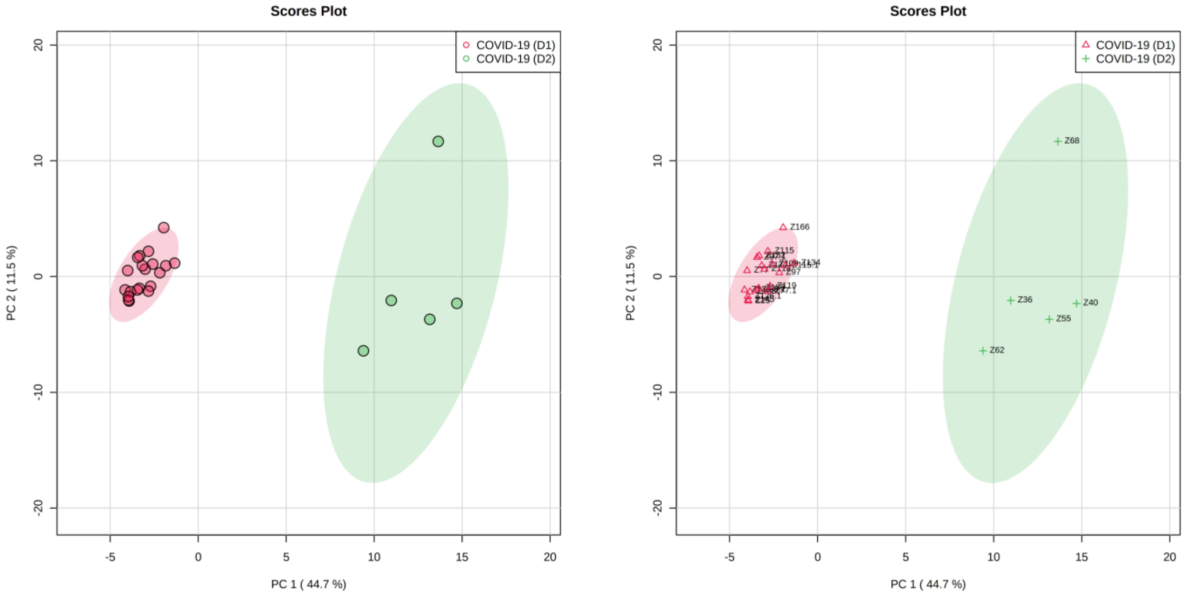

Figure S2. Two-dimensional principal component analysis (2D PCA) scores plot of the relative phospholipid content in the plasma of 15 deceased COVID-19 patients **[COVID-19 (D1)]** and another group of 5 deceased COVID patients **[COVID-19 (D2)]**.

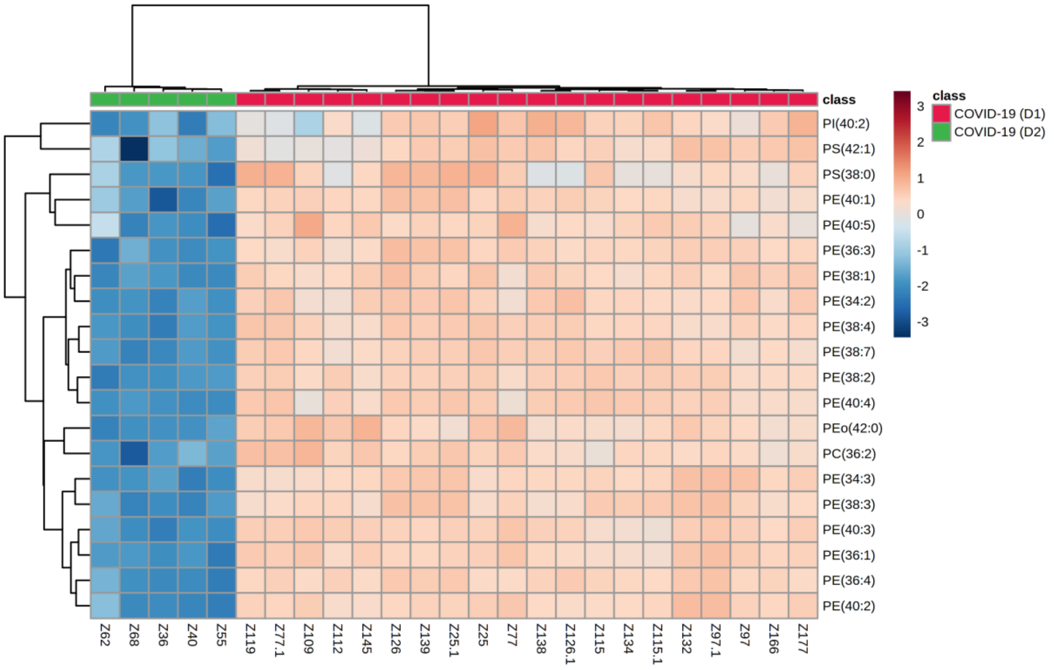

Figure S3. Two-dimensional hierarchical clustering heat map of the 20 most discriminating phospholipid species (according to One-way ANOVA) identified in the plasma of 15 deceased COVID-19 patients **[COVID-19 (D1)]** and another group of 5 deceased COVID patients **[COVID-19 (D2)]**.

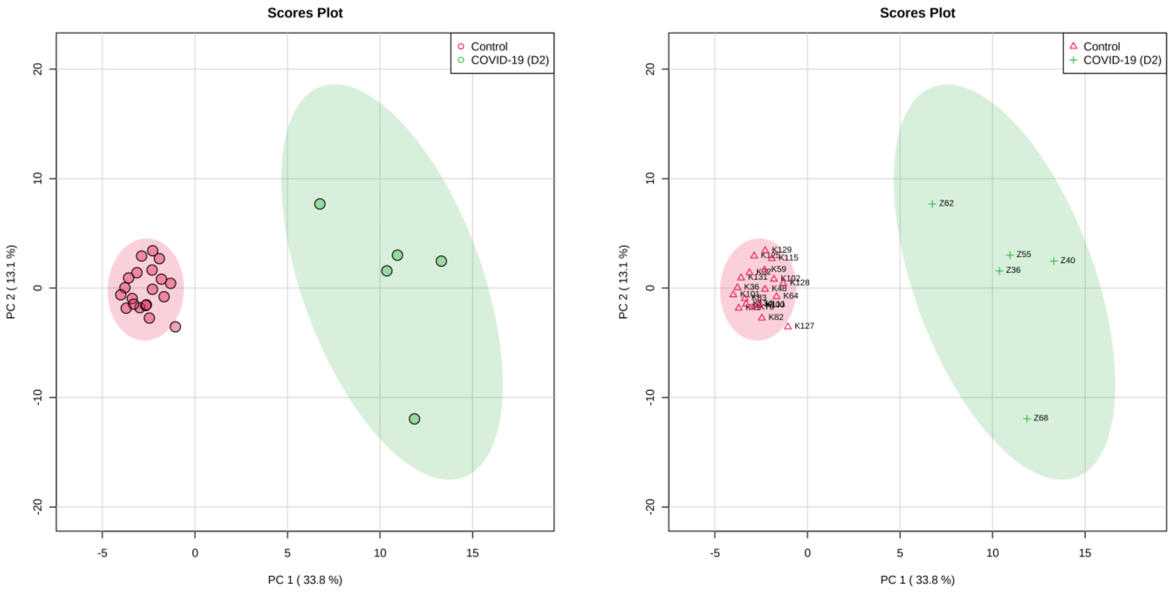

Figure S4. Two-dimensional principal component analysis (2D PCA) scores plot of the relative phospholipid content in the plasma of 20 healthy subjects **[Control]** and 5 deceased COVID patients **[COVID-19 (D2)]**.

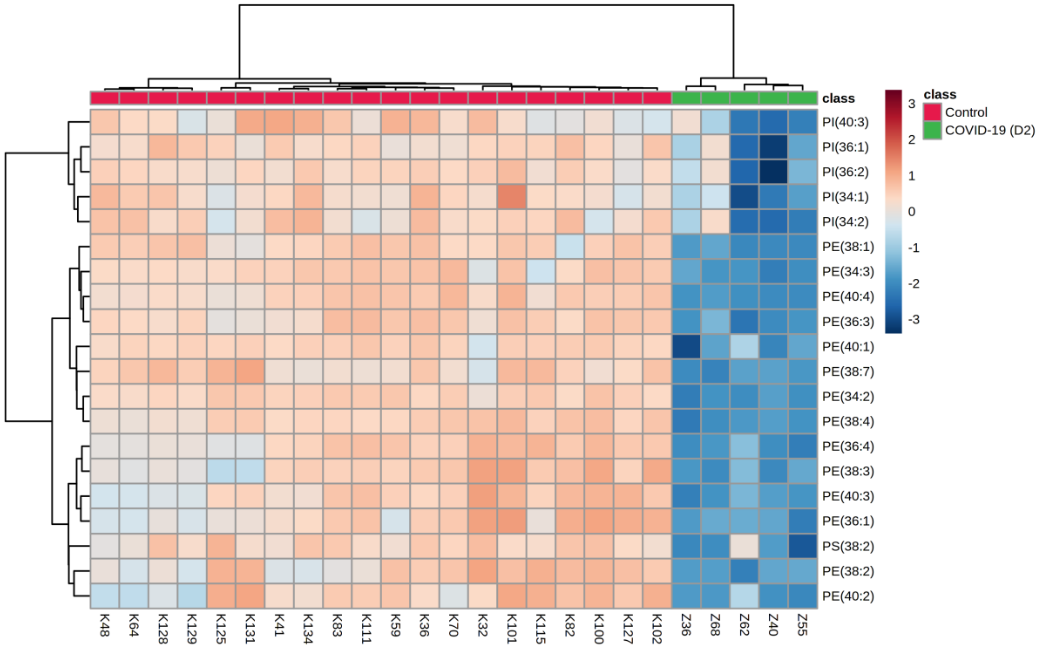

Figure S5. Two-dimensional hierarchical clustering heat map of the 20 most discriminating phospholipid species (according to One-way ANOVA) identified in the plasma of 20 healthy subjects **[Control]** and 5 deceased COVID patients **[COVID-19 (D2)]**.
